# A Review on Pipeline In-Line Inspection Technologies

**DOI:** 10.3390/s25154873

**Published:** 2025-08-07

**Authors:** Qingmiao Ma, Weige Liang, Peiyi Zhou

**Affiliations:** Naval University of Engineering, Wuhan 430033, China; m24182607@nue.edu.cn (Q.M.); d23382604@nue.edu.cn (P.Z.)

**Keywords:** pipeline in-line inspection technology, electromagnetic testing, acoustic testing, optical testing, robotic technology

## Abstract

Pipelines, as critical infrastructure in energy transmission, municipal facilities, industrial production, and specialized equipment, are essential to national economic security and social stability. This paper systematically reviews the domestic and international research status of pipeline in-line inspection (ILI) technologies, with a focus on four major technological systems: electromagnetic, acoustic, optical, and robotic technologies. The operational principles, application scenarios, advantages, and limitations of each technology are analyzed in detail. Although existing technologies have achieved significant progress in defect detection accuracy and environmental adaptability, they still face challenges including insufficient adaptability to complex environments, the inherent trade-off between detection accuracy and efficiency, and high equipment costs. Future research directions are identified as follows: intelligent algorithm optimization for multi-physics collaborative detection, miniaturized and integrated design of inspection devices, and scenario-specific development for specialized environments. Through technological innovation and multidisciplinary integration, pipeline ILI technologies are expected to progressively realize efficient, precise, and low-cost lifecycle safety monitoring of pipelines.

## 1. Introduction

In contemporary society, pipelines, as critical infrastructure in energy transmission, municipal facilities, industrial production, and specialized equipment, directly impact national economic stability and social security through their safety and reliability. In the energy sector, long-distance oil and gas pipelines, responsible for transporting vital national energy resources, may result in major safety incidents if failure occurs. For municipal systems, leakage or blockage of water supply and drainage pipelines can lead to functional paralysis of urban operations; in industrial scenarios, defects in process pipelines may trigger production shutdowns or environmental disasters [1,2,3]; for specialized equipment (e.g., artillery barrels, aviation hydraulic pipelines), pipeline damage compromises equipment performance and personnel safety [4,5].

**Figure 1:**
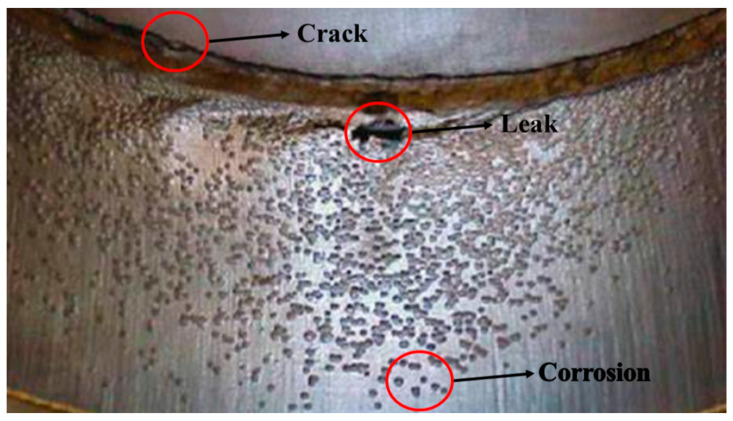
Damage inside the oil and gas pipeline.

Pipelines are persistently subjected to complex operating conditions—including high temperature, high pressure, corrosive media, and mechanical stress—leading to defect such as cracks, corrosion, and deformation, as illustrated in Figure 1. Traditional manual inspection methods suffer from low efficiency, elevated operational risks, and blind zone coverage limitations. In contrast, in-line inspection (ILI) technologies utilize sensor-equipped robotic devices integrated with intelligent algorithms to perform internal pipeline assessments, enabling non-contact, high-precision defect monitoring. This paper systematically examines the operational principles, practical applications, and technical challenges of four core ILI methodologies: electromagnetic, acoustic, optical, and robotic detection technologies. By prospectively analyzing future development trajectories, the study aims to provide a theoretical foundation for advancing pipeline integrity management and safety assurance strategies.

## 2. Research Status

With the diversification of pipeline applications and the increasing demand for inspection technologies, researchers have established four core technological frameworks centered on electromagnetic, acoustic, optical, and robotic systems through systematic studies on pipeline ILI. These technologies, grounded in distinct physical principles, offer tailored solutions for metallic and non-metallic pipelines, diverse defect types (e.g., corrosion, cracks, deformation), and complex environments (high-temperature, multiphase flow, confined spaces). Academia and industry have achieved continuous breakthroughs in enhancing detection accuracy, improving environmental adaptability, and reducing equipment costs, while simultaneously exploring novel pathways for multi-technology collaboration and intelligent algorithm integration. This section systematically reviews the research progress of each detection technology from three perspectives: technical principles, representative applications, and existing challenges, thereby elucidating their technological characteristics and developmental trends.

### 2.1. Electromagnetic Inspection Technologies

Electromagnetic inspection technologies identify defects through interactions between electromagnetic fields and pipeline materials, primarily including Magnetic Flux Leakage (MFL), Eddy Current Testing (ECT), and Pulsed Eddy Current Testing (PECT) [6].

**Figure 2:**
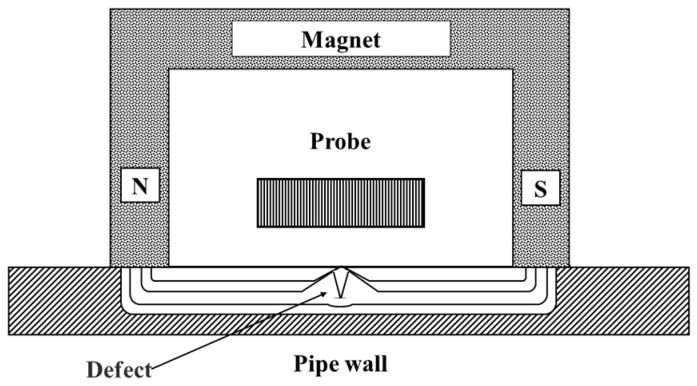
MFL Testing.

MFL technology utilizes permanent magnets to establish a magnetic field within a pipeline. When defects are present in the pipeline wall, the magnetic field distribution becomes distorted. Defects are identified by detecting changes in the leakage magnetic field [7]. Differentiation between internal and external damage is achieved through the analysis of the time-domain characteristics and energy-level classification of the leakage field [8], the differential transformation of MFL signals coupled with single-point signal redundancy detection [9], the tangential and normal components of the leakage magnetic field [10], and the magnetic flux concentration detection method [11]. A schematic representation of the inspection principle is shown in Figure 2. The core technologies underpinning MFL inspection encompass high-sensitivity magnetic sensors, optimized magnetic circuit design, and data acquisition and processing algorithms [12].

Based on this technology, Shilpi Saha et al. developed a novel detection tool that is extremely lightweight and can be easily moved axially and circumferentially along pipelines [13]. Dong Shaohua et al. implemented MFL technology in oil and gas pipelines, developing ultra-high-definition (UHD) MFL internal inspection technology. This advancement enables detailed defect characterization, effectively addressing the challenge of detecting pinhole-sized localized deep corrosion in oil and gas pipelines and enhancing defect quantification capabilities [14]. Qi Guangfeng et al. investigated internal inspection technologies for subsea pipelines, highlighting the limitations of MFL technology in pipelines with complex geometries, significant internal deposits, or poor flow conditions [15]. Russell D. Morris et al. addressed the effects of hydrogen embrittlement in hydrogen pipelines on MFL technology, discussing technical challenges and future directions [16].

Nevertheless, MFL inspection technology exhibits limitations in identifying complex defects (e.g., overlapping defects), is susceptible to interference from the pipeline surface condition, and suffers from the low efficiency and high misjudgment rates associated with traditional manual analysis [17].

**Figure 3:**
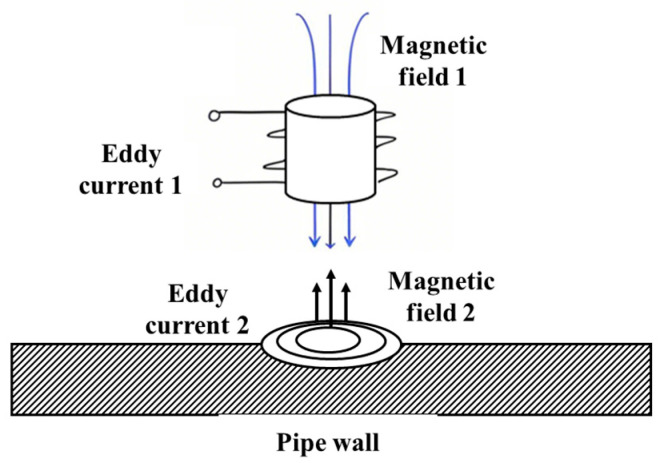
ECT.

ECT technology relies on the interaction between an alternating electromagnetic field and a conductive material to achieve high-sensitivity identification of near-surface defects [18], as illustrated schematically in Figure 3. This technique exhibits equal sensitivity to defects on both the internal and external walls of a pipeline. However, the identification of internal wall defects can be achieved by enhancing the resolution of the detection coil [19].

M.F. Abdul Halim et al. proposed a portable ECT method which demonstrated agreement with ultrasonic testing results in experimental comparisons [20]. Lu Yading et al. designed a planar L-shaped eddy current sensor capable of effectively detecting defects in all orientations. This addresses the directional specificity limitation inherent in conventional sensors and enhances the capability for detecting subsurface defects in metallic oil and gas pipelines [21]. Chen Shukai et al. utilized ECT to quantitatively measure the carburization thickness in ethylene cracking furnace tubes, based on the material’s phase transformation from paramagnetic to ferromagnetic properties [22]. Dong Shihao et al. designed an online array eddy current inspection system for high-speed production lines to meet the demands for rapid defect detection and identification of small flaws [23].

However, ECT technology suffers from a limited penetration depth and is inapplicable to non-metallic pipelines [24]. Further refinement of multi-frequency eddy current techniques is required [25].

PECT Technology applies pulsed current to a conductor to generate a pulsed magnetic field. Defects are detected by analyzing the decay characteristics of the induced eddy currents [26]. The location of defects can be determined based on the waveform of the differential conductivity [27].

Changxin Wang et al. conducted numerical simulations of PECT for crack detection, analyzing the correlation between lift-off, time frequency signals, and crack width and depth [28]. Yang Chao et al. aimed to improve the signal-to-noise ratio (SNR) of transient response signals in PECT and achieve effective detection of pipeline defects. By modifying detection coil parameters, they enhanced detection sensitivity [29]. Tao Aijun et al. addressed the sensitivity issue in PECT. Through a combination of numerical simulation and experimentation, they investigated the influence of sensor positioning on sensitivity [30]. Wang Jin et al. proposed an external PECT method based on dual magnetic field sensing for the quantitative inspection of defects in in-service oil well tubing. This method enables the three-dimensional reconstruction of corrosion defects on the inner wall of the tubing and demonstrates high accuracy [31].

However, PECT technology currently lacks robustness in the quantitative analysis of complex defects and is susceptible to electromagnetic interference (noise) [32].

### 2.2. Acoustic Inspection Technologies

Acoustic inspection technologies achieve defect localization by analyzing acoustic wave propagation characteristics, primarily including Ultrasonic Testing (UT) and Acoustic Emission Testing (AET).

**Figure 4:**
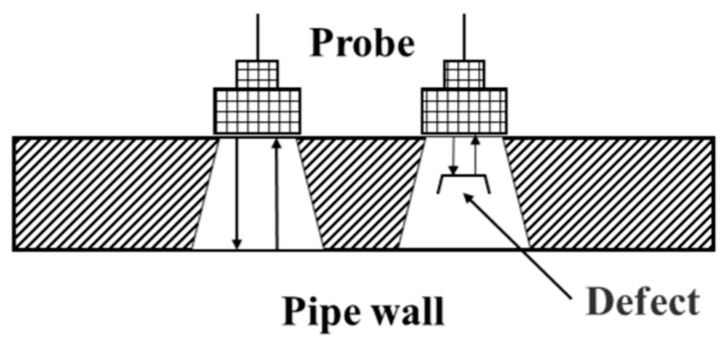
UT.

UT technology utilizes the propagation characteristics of ultrasonic waves within a medium. Defects and their locations are detected by measuring parameters of the reflected waves, such as time-of-flight and amplitude [33], as schematically shown in Figure 4.

Cao Jianshu et al. employed laser ultrasonic technique to detect internal surface cracks in X80 pipelines, highlighting the issues of high equipment cost and complex operation [34]. Martin H. Skjelvareid et al. employed the virtual source method with synthetic aperture focusing to extend the effective range of a monolithic transducer in focused ultrasound and enhance its lateral resolution [35]. Yang Peng investigated the capability of ultrasonic guided waves for inspecting complex structures (e.g., bends, welds), observing that signal attenuation adversely affects accuracy [36]. Jun Okamoto Jr et al. developed an ultrasonic pig equipped with 16 transducers, featuring an onboard energy system along with data acquisition and storage systems [37]. Zaid A. Mehd et al. proposed a flexible, non-contact, fully coil-based electromagnetic acoustic transducer (EMAT) designed to accommodate a range of diameters, suitable for smooth surfaces or pipes where most surfaces are coated [38].

However, UT requires the use of a couplant, is sensitive to surface roughness, and its inspection speed is limited [39].

**Figure 5:**
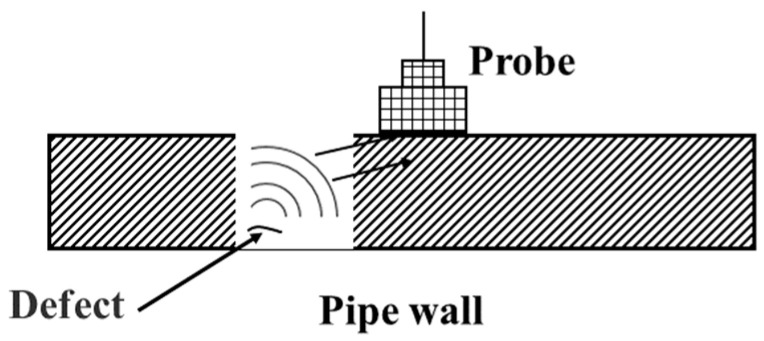
AET.

AET Technology detects the location of pipeline cracks or leaks by capturing the elastic waves released during material deformation or fracture using sensors and analyzing signal characteristics such as amplitude, frequency, and energy [40], as schematically illustrated in Figure 5.

Yang Lei et al. simulated three types of acoustic sources on pipelines—lead-breaking, metal rod impacts, and sandpaper friction. After signal acquisition, they employed wavelet denoising to extract effective features and compared time-domain and frequency spectrum differences to identify fault types. This work remained a laboratory simulation and did not address practical application in complex noise environments [41]. Kosuke Yoshida et al. conducted time-domain analysis of microwaves to facilitate non-destructive testing of pipelines using electro-optic sensors [42]. Zhang Haoqi et al., addressing the need for online inspection of boiler main steam pipelines and the difficulties of conventional methods, proposed an approach utilizing AE sensors connected via waveguide rods and verified its feasibility [43]. Bai Chundong et al. effectively detected corrosion defects in gas transmission pipelines by studying AE signal types, frequency spectrum characteristics, and energy distribution [44].

However, AE testing technology suffers from several limitations: complex signal processing requirements, susceptibility to interference from external environmental noise, localization accuracy adversely affected by dispersion effects, difficulty in detecting multi-point leaks, and severe signal attenuation over long distances [45].

### 2.3. Optical Inspection Technologies

Optical inspection technologies are characterized by non-contact operation and high precision, encompassing machine vision, laser scanning, and distributed fiber optic sensing (DFOS).

**Figure 6:**
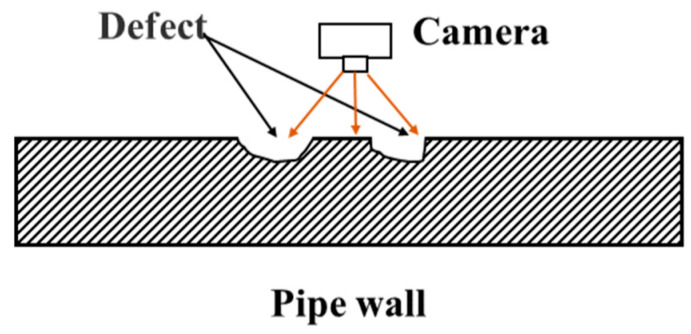
Machine vision inspection technology.

Machine vision inspection technology enables automated defect recognition based on high-resolution image acquisition and image recognition algorithms, achieved through feature extraction and classifier design [39], as schematically illustrated in Figure 6.

Huang Mao improved the Mask Region-based Convolutional Neural Network (Mask R-CNN) algorithm to detect defects on the inner surface of polyethylene pipelines [46]. Li Jiangtao et al. employed the YOLOv7-tiny algorithm for detecting cracks on the inner surface of gas pipelines [47]. Ruihao Liu et al. proposed the Sewer-YOLO-Slim model combined with 3D reconstruction. This approach not only enables the detection of defects in urban sewer pipelines, but also facilitates three-dimensional reconstruction of the defects, achieving quantitative inspection [48]. Mingcun Liu et al. utilized partial convolution image inpainting combined with Histogram of Oriented Gradient (HOG) features and a Support Vector Machine (SVM) classifier for processing drainage pipeline defect detection [49]. Xinyu Shang et al. enhanced the robustness of drainage pipeline defect detection by fusing handcrafted features with Visual Geometry Group (VGG) network features [50].

However, the performance of machine vision inspection technology degrades under low-light or complex environmental conditions [51]. Optimization of image enhancement techniques and refinement of algorithms are required to address these limitations [52].

**Figure 7:**
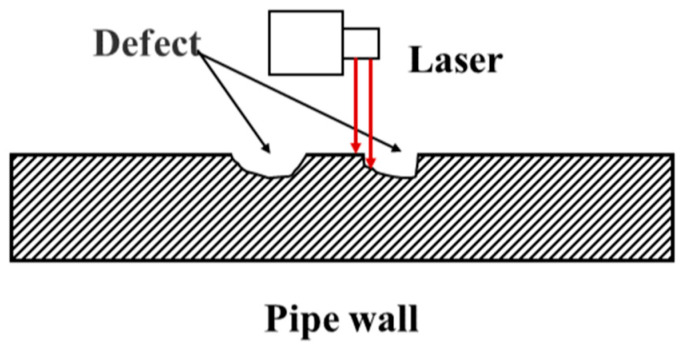
Laser inspection technology.

Laser inspection technology utilizes laser beams to scan the inner wall of pipelines. Geometric shape and defect information are acquired by measuring the reflected laser signals [53,54], as schematically illustrated in Figure 7.

Sun Yi et al. collected inner wall point cloud data using a pipeline laser scanning device. By applying a fast Iterative Closest Point (ICP) algorithm accelerated through a k-nearest neighbor search based on adaptive spatial spheres, they obtained an intuitive and stereoscopic 3D contour map of the inner pipeline wall, enabling quantitative defect detection [55]. Zhou Jia combined structured light centerline extraction with a point cloud simplification algorithm to achieve general pipeline defect detection, although this approach exhibits poor adaptability to low-reflectivity surfaces [56]. Wang Yongtao et al. utilized a solid-state area-array LiDAR (Light Detection and Ranging) system for inspecting drainage pipelines, noting the need for enhanced anti-interference capabilities [57]. Weslley Silva et al. conducted comparative measurements among three techniques—digital detector arrays (DDAs), coplanar translation tomography, and computed tomography (CT)—for their application in composite pipeline inspection [58].

However, laser inspection technology suffers from relatively high equipment costs and poor adaptability to low-reflectivity surfaces [59].

DFOS technology utilizes optical fiber as the sensor to monitor pipeline parameters such as strain and temperature by detecting changes in the optical signal propagating through the fiber. This enables the detection and localization of defects [60].

Qiu Xiufen et al. detected leaks in oil and gas pipelines by combining short-time average energy with a modified zero-crossing rate [60]. Yin Hailong et al. employed distributed temperature sensing (DTS) based on optical fiber combined with a wavelet denoising algorithm to inspect sewage pipelines [61].

However, the complexity of fiber installation and concerns regarding long-term stability necessitate further validation, requiring intensified field trials [62].

### 2.4. Robotic Technology

Robotic technology enables autonomous in-pipe inspection through sensor-equipped systems, primarily categorized into wheeled/tracked robots and continuum robots [63]. Wheeled/tracked pipeline inspection robots are designed with wheel/track propulsion mechanisms, propelling along internal walls to provide enhanced mobility and adaptability in regular-diameter pipeline environments, while carrying sensors for real-time defect assessment [64].

Zhang Wentao et al. proposed an integrated approach combining ultrasonic phased array and microwave detection technologies for inspecting urban polyethylene gas pipelines [65]. Hidemasa Sawabe et al. proposed a control method for articulated wheeled robots traversing straight pipes, bends, and branches, enabling inspection of extended areas [66]. Mao Liuwei et al. developed a wheeled robot and utilized ADAMS software simulation to optimize detection stability in small-diameter pipelines [67]. Zang Yanxu et al. devised a single-arm-supported probe mechanism adaptable to varying wall thicknesses in small-diameter pipelines, albeit with constrained cable routing flexibility [68]. Ding Rong et al. deployed an acoustic detector mounted on a robot for application in refined oil pipelines; however, the detection range was significantly affected by environmental noise, and sensitivity to small defects was relatively low [69].

Nevertheless, the locomotion efficiency and adaptability of existing robotic technologies within complex pipeline networks (e.g., those featuring multiple bends or diameter transitions) still require further optimization [70]. Enhancements in obstacle negotiation capability and operational endurance are essential. Wheeled robots exhibit limited traction with the pipe wall due to friction constraints, rendering them unsuitable for internal inspection of oil and gas pipelines [71]. Tracked robots suffer from increased structural complexity, greater control challenges, larger size, and compromised maneuverability [72].

Continuum robots, mimicking the flexible structures of biological organisms, navigate through pipelines via pneumatic or hydraulic actuation, carrying sensors for inspection [73].

Fu Hai et al. developed an all-terrain amphibious robot adaptable for inspecting pipelines with high water levels and sediment accumulation, though it exhibits high cost and requires endurance optimization [74]. Devesh Mishra et al. developed a robot capable of autonomous locomotion within crude oil pipelines by harnessing the kinetic energy of flowing oil for propulsion in buried pipelines [75]. Mostafa A. Atalla et al. developed a mechanically inflatable bio-inspired robot capable of adapting to pipelines of varying sizes and shapes; however, its locomotion speed is relatively slow [76]. Li Wenzhang et al. created a fully pneumatic pipeline inspection robot, whose operation relies on a compressed air source, consequently limiting its environmental adaptability [77]. Rakiba Rayhana et al. proposed a deep learning-based autonomous inspection framework to ensure continuous detection and facilitate robotic navigation [78].

Future enhancements require integrating bio-inspired designs with advanced lightweight materials to improve locomotion agility through design optimization and control advancements, including robotic navigation [79] and related domains [80].

### 2.5. Multi-Technology Integration and Special Applications

Multi-technology collaborative inspection enhances defect identification accuracy by fusing physical fields with data algorithms. Physical field fusion integrates and processes data from multiple sensors, performing comprehensive processing and optimization of various collected data. It automatically performs fusion processing—including detection, association, correlation, and estimation—of information from multiple sources, thereby enhancing the overall system’s accuracy and robustness [81]. Multi-sensor data fusion operates at three distinct levels, processing information to varying degrees: Data-Level Fusion, Feature-Level Fusion, and Decision-Level Fusion [82]. These levels impose different requirements on the data and employ distinct processing methodologies.

**Figure 8:**
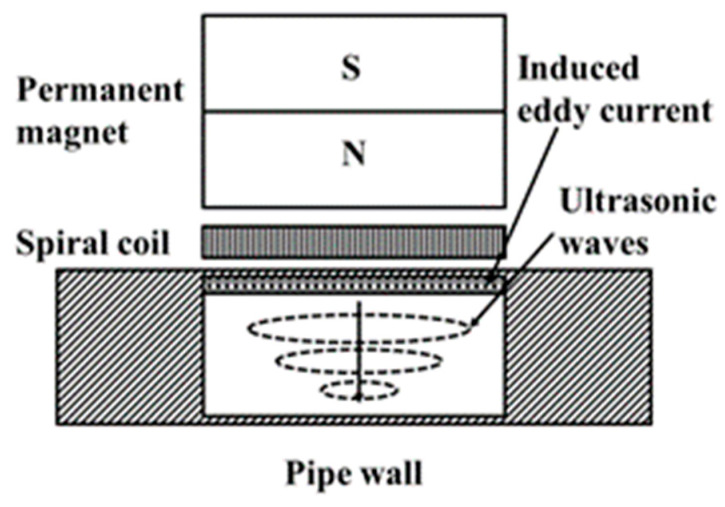
PECT-EMAT Composite Inspection Method.

Ou Zhengyu et al. analyzed existing buried pipeline inspection technologies and proposed integrating multiple detection techniques to form a comprehensive evaluation system. This multi-technology synergy enhances inspection accuracy and reliability [83]. Cao Jianshu et al. designed a LabVIEW-based laser ultrasonic system for gas pipeline inspection. This system improves the spatial resolution of traditional ultrasonic testing and enables the rapid detection of various defects in oil and gas pipelines [84]. Zhou Xiantong et al. utilized laser ultrasonic visualization technology to analyze the dynamic propagation process of echo waves, enabling the direct visual detection of defects inside bent pipes and determining defect locations based on propagation characteristics [85]. Ali Ahmadian Mazraeh et al. integrated ultrasonic and eddy current sensors onto a variable-diameter robot platform, significantly enhancing detection accuracy and precision [86]. Wang Chao et al. targeting the simultaneous detection of internal and external defects, proposed an integrated Pulse Eddy Current Electromagnetic Acoustic Transducer (PECT-EMAT) composite inspection method. This approach benefits from the high physical compatibility of the two techniques, offering advantages over traditional multi-system parallel solutions by improving inspection efficiency and reducing costs. However, the signals are susceptible to noise interference. A schematic diagram of this detection method is shown in Figure 8 [87]. Sun Xianrui integrated periscope inspection technology with Geographic Information System (GIS) for urban drainage pipeline inspection, though its effectiveness depends heavily on the accuracy of video recognition algorithms [88]. Wu Di et al. analyzed existing drainage pipeline inspection robot products and proposed combining video inspection with radar detection methods; however, their adaptability in complex environments requires further validation [89]. Alexander G. Rumson et al. proposed an unmanned automated inspection methodology integrating hardware and software, utilizing paired autonomous underwater vehicles (AUVs) [90] and unmanned surface vehicles (USVs) for subsea pipeline examination [91]. Claudio Passucci et al. engineered a novel compact multi-parameter pipeline inspection tool deployable standalone or robotic-assisted, enabling system control and data acquisition [92]. Zeng Weiguo et al. researched non-contact magnetic stress detection, indicating that its grading standards require optimization [93].

Future development necessitates creating a universal multi-physics field inspection platform, tailoring solutions for extreme environments, and integrating digital twin technology to achieve full lifecycle monitoring [94].

In the field of special equipment inspection, Han Chao et al. developed a laser circumferential scanning technique to achieve high-precision inspection of gun barrel inner bores [4]. Zhang Jin et al. utilized electromagnetic acoustic surface-horizontal shear waves (SAWs) to detect cracks in chamber bores [5]. Yingxiu Cao et al. implemented machine vision image stitching technology for gun barrel defect detection [95]. Zhao Kai employed laser inspection combined with 3D reconstruction to assess inner bore wear [96]. Lu Zhuo et al. adopted laser displacement measurement technology for detecting surface flaws in inner bores [97]. Chang Jingfeng enhanced rifling dimensional accuracy through integrated laser visual composite inspection [98]. However, technologies in this domain exhibit high specialization and limited scalability, with research often constrained by its classified nature and restricted public discourse.

In summary, the advantages and limitations of existing pipeline in-line inspection (ILI) technologies are comprehensively compared in Table 1. Their capabilities in quantitative and qualitative defect analysis are visually summarized in Table 2. Future development should focus on domain-specific technological complementarity, where the simultaneous application of multiple techniques enables efficient, accurate, and cost-effective pipeline inspection.

## 3. Challenges and Future Perspectives

### 3.1. Current Challenges

Comparative analysis of existing pipeline in-line inspection technologies, as summarized in Table 3 (Technology Applicability Scope), reveals significant operational challenges in practical applications, where complex environmental conditions impose severe constraints on technical adaptability; pipelines routinely operating under extreme service conditions—including elevated temperatures, high humidity, and multiphase flow media—induce sensor performance degradation, exemplified by thermal drift in magnetic sensors reducing MFL measurement accuracy under high-temperature environments, and severe signal interference compromising acoustic-based detection in high-noise scenarios.

Furthermore, specialized scenarios such as high-pressure corrosive conditions in subsea pipelines impose heightened demands on equipment material resilience and sealing integrity, where the fundamental trade-off between inspection precision and operational efficiency remains unresolved. High-accuracy techniques like laser scanning rely on dense point cloud data, incurring substantial computational overhead and reduced inspection speed; conversely, rapid methods such as *MFL* exhibit operational efficiency but suffer from insufficient sensitivity to micro-defects and limited capability in identifying overlapping defects. Real-time processing capabilities for massive inspection data are inadequate—exemplified by the computational complexity of 3D reconstruction models and the high false-positive rates of video analytics under complex lighting. Ultimately, prohibitive equipment costs and specialized applicability constrain technological dissemination: advanced systems like continuum robots and distributed fiber sensing require significant R&D investment, rendering them financially prohibitive for small-to-medium enterprises (SMEs), while specialized apparatuses (e.g., artillery barrel inspectors) demonstrate poor scalability to civilian applications due to domain-specific design constraints.

### 3.2. Future Directions

**Figure 9:**
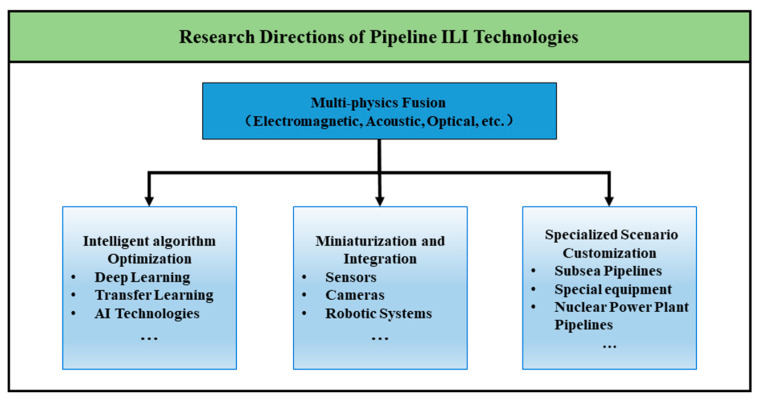
Future Research Directions for Pipeline ILI Technologies.

Future research in pipeline in-line inspection technologies should focus on multi-physics field fusion for comprehensive detection, integrating electromagnetic, acoustic, and laser-based multidimensional data to transition defect analysis from single-parameter evaluation to holographic characterization [99], as specifically illustrated in Figure 9.

**Figure 10:**
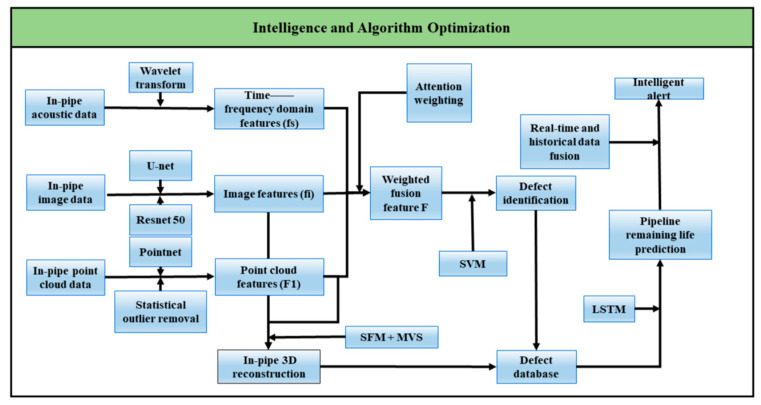
Technical Pathway for Intelligent Algorithm Optimization.

The first approach focuses on intelligent algorithm optimization, where multimodal feature fusion enhances defect recognition capability, enabling rapid, efficient, and real-time detection [100]. This methodology leverages data-driven model refinement to achieve comprehensive defect identification, detection, reconstruction, and prediction, as specifically illustrated in Figure 10.

For multimodal inspection tasks in pipeline interiors, a systematic data processing and analysis pipeline must be established. First, preprocessing and feature extraction perform on acoustic, image, and point cloud data. Acoustic signals undergo wavelet transform for environmental noise removal, extracting time-frequency domain features (Fs) including short-time energy spectral density and Mel-frequency cepstral coefficients to quantify material thickness variations and acoustic response characteristics of internal defects [101]. Image data achieves pixel-level segmentation via U-Net to distinguish corrosion regions from cracks, while ResNet-50 extracts deep features (Fi), with attention mechanisms prioritizing critical areas like weld seams [102]. Point cloud data applies statistical filtering to eliminate outliers, then employs PointNet to extract geometric features (Fl) including curvature and surface roughness, accurately characterizing 3D deformations [103].

Secondly, defect diagnosis is achieved through cross-modal feature fusion and model training. A Transformer module [104] based on multi-head attention mechanism is designed to dynamically weight and fuse acoustic features (Fs), image features (Fi), and point cloud features (Fl), generating a 1024-dimensional comprehensive feature vector that is input into a Support Vector Machine (SVM) [105] for defect classification. Concurrently, high-precision dense point clouds are reconstructed from multi-view images using Structure from Motion (SFM) [106] and Multi-View Stereo (MVS) [107] algorithms to restore the three-dimensional structure of pipeline inner walls. A structured defect database is constructed to archive historical data (including defect typology, dimensional parameters, and environmental variables), while implementing a relational database framework with optimized indexing mechanisms to enable real-time similarity-based case retrieval [108]. This architecture enhances online diagnostic efficiency.

Finally, the operational strategy is enhanced through temporal sequence analysis and intelligent decision-making frameworks. Long Short-Term Memory (LSTM) networks are employed to model defect dimension time-series data, enabling prediction of future propagation trends [109]. By integrating real-time inspection results with historical database records, the system automatically generates maintenance recommendations.

**Figure 11:**
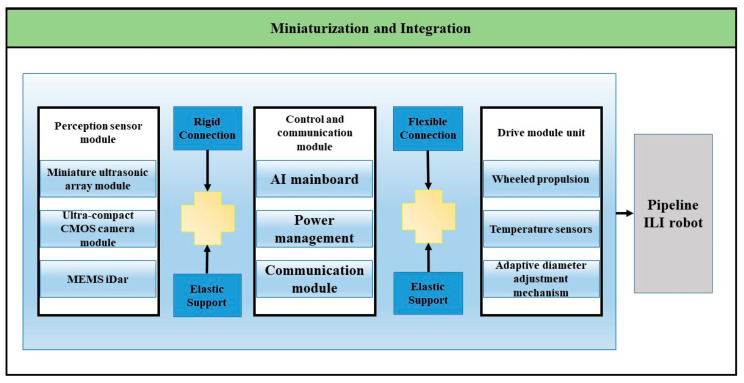
Technological Pathway for Miniaturization and Integration.

The second approach focuses on miniaturization and integration, employing micro-sensors with modular designs that are deployed on robotic platforms for inspection tasks, as specifically illustrated in Figure 11.

The hardware architecture of pipeline ILI robot is designed based on the principles of multimodal sensor integration and modular functional partitioning. The perception sensor module, serving as the core sensing unit, integrates a miniature ultrasonic module, an ultra-compact CMOS camera module, and a MEMS solid-state LiDAR to achieve pipeline wall thickness measurement, visual detection of internal surface defects, and high-precision 3D morphology reconstruction, respectively. All sensors achieve spatiotemporal alignment through hardware-synchronized triggering circuits, minimizing temporal synchronization errors to ensure spatiotemporal consistency in multi-source data fusion [110].

The control and communication module functions as the central hub, integrating an AI [111] mainboard responsible for real-time processing and fusion algorithm execution of multimodal data; the power management system adopts a dual-redundant design, with primary power supplied by lithium -polymer batteries and supplemented by supercapacitor-based emergency power; the communication module delivers transmission bandwidth meeting uplink requirements and fulfills low-latency backhaul demands for video streams and point cloud data.

The tail functional unit incorporates a wheeled propulsion module, adaptive pipe-diameter adjustment mechanism (lead screw-linkage assembly), and high-precision temperature sensors, achieving adaptive locomotion in complex pipeline environments through closed-loop control algorithms.

The robot’s perception-control module and control-communication module are interconnected via a flange-based rigid connection, coupled with a dowel pin positioning structure, ensuring attitude stability of the sensor module during high-speed motion; the interface between the control-communication module and drive module unit employs a flexible hinge connection, enabling tail deflection during navigation through pipe bends.

The third approach involves customized design for extreme operating conditions, requiring the establishment of multi-scale simulation models tailored to specific scenarios. For underwater pipelines characterized by high pressure, strong corrosivity, and low visibility, the robot’s customized design must incorporate multi-scale simulation and parameter self-adaptation optimization. Through coupled simulations modeling underwater mechanical stress distribution, the pressure-resistant hull is optimized to balance compressive strength and lightweight requirements. Algorithmically, dynamic multi-modal parameter adaptation is implemented for complex environments: The acoustic module employs adaptive wavelet threshold denoising [112]; the vision system integrates underwater scattering compensation algorithms with dark channel prior and bilateral filtering to enhance structural similarity [113]; cameras achieve crack detection in turbid water; LiDAR is optimized for blue-green wavelengths with time-of-flight dynamic calibration [114]; and data fusion utilizes cascaded attention mechanisms to dynamically weight acoustic, visual, and point cloud features based on real-time visibility.

## 4. Conclusions

Significant advancements have been achieved in pipeline ILI technologies across electromagnetic, acoustic, optical, and robotic domains. Techniques such as MFL detection, ultrasonic guided waves, laser scanning, and multi-sensor robotic systems enable defect identification and 3D reconstruction. Nevertheless, the engineering application of these technologies still faces core challenges, including multiphysics coupling dissonance, conflicts in cross-scale inspection paradigms, delays in holistic data fusion, and lifecycle cost control. Future breakthroughs will necessitate focusing on adaptive sensing mechanisms in complex environments (specifically electromagnetic- acoustic-thermomechanical field co-reconstruction), a digital twin decision-making architecture empowered by artificial intelligence (enabling a real-time detection-identification-assessment closed loop), and a reconfigurable modular technological ecosystem (optimizing the design-manufac-uring-service value chain). This integrated approach will drive the evolution of pipeline monitoring systems towards an intelligent, collaborative paradigm characterized by self-perception, self-decision-making, and self-evolution, thus providing scientific support for ensuring pipeline safety throughout the entire lifecycle and establishing a digitalized and intelligent foundation for the intrinsic safety of pipeline transportation systems.

## Figures and Tables

**Table 1:** Advantages and Limitations of Pipeline ILI Technologies.

Technology	Advantages	Disadvantages
MFL	High efficiency for volumetric defects in ferromagnetic pipes; Strong contamination resistance; Suitable for large-diameter pipelines	Limited to ferromagnetic materials; Low sensitivity to axial cracks; Requires measurable wall loss
ECT	High-precision surface crack detection; No couplant required; Rapid response	Limited to conductive materials; Shallow penetration depth; Susceptible to lift-off effects
PECT	Capable of inspecting through insulation layers; Non-removal of coatings; Non-contact	Restricted to carbon steel/ferromagnetic materials; Low resolution; Vulnerable to electromagnetic interference
UT	Accurate thickness measurement; Internal defect detection; Applicable to multi-material pipes	Requires coupling medium; Highly affected by surface roughness; Limited in high-temperature environments
AET	Real-time dynamic monitoring; Large coverage; No active scanning required	Detects only active defects; poor localization accuracy; Noise interference susceptibility
Machine Vision	Direct visualization of defects; High-resolution imaging; Comprehensive digital records	Dependent on illumination/surface cleanliness; Lens contamination vulnerability; Significant blind spots
Laser	Sub-millimeter 3D mapping; Non-contact; High-precision corrosion quantification	Extremely high cost; Requires reflective surfaces; Slow scanning speed
DFOS	Long-distance real-time monitoring; Multi-parameter measurement (temp/vibration/strain); EMI immunity	Low spatial resolution (meter-level); Insensitive to micro-leaks; Complex installation
Wheeled/ Tracked Robots	High payload capacity; Extended operation time; Suitable for straight/gentle-bend pipes	Poor obstacle negotiation (e.g., tees/reducers); Limited terrain adaptability; Prone to jamming
Continuum Robots	Exceptional maneuverability (elbows/valves); ideal for small-diameter/complex networks	Extremely high cost; Complex control; Short endurance; Limited tether length

**Table 2:** Quantitative and Qualitative Analysis Capabilities of Pipeline ILI Technologies.

Technology	Quantitative Defect Analysis Capability	Qualitative Defect Analysis Capability
MFL	Yes (Wall loss, corrosion depth)	Limited (Volumetric defects only)
ECT	Semi-quantitative (Crack length/depth estimation)	Yes (Crack/pitting classification)
PECT	Yes (Average wall thinning rate)	No (Defect type identification unavailable)
UT	Yes (Wall thickness ±0.1 mm, defect sizing)	Yes (Internal/external defect differentiation)
AET	No (Intensity grading only)	Yes (Defect activity typification)
Machine Vision	No (Requires calibration)	Yes (Surface defect morphology identification)
Laser	Yes (3D topography)	Yes (Corrosion/deformation pattern discrimination)
DFOS	Yes (Temperature/strain quantification)	Yes (Event-type recognition: leaks/third-party damage)
Wheeled/Tracked Robots	Contingent on integrated sensors	Contingent on integrated sensors
Continuum Robots	Contingent on integrated sensors	Contingent on integrated sensors

**Table 3:** Application Scope of Pipeline ILI Technologies.

Technology	Application Scope	Limitations
MFL	Ferromagnetic pipelines (oil/gas/water); volumetric defects (pits, wall thinning).	Ferromagnetic materials only; insensitive to axial cracks; requires clean surfaces; speed-dependent accuracy.
ECT	Surface defects in conductive pipelines (petrochemical/nuclear); cracks, pitting corrosion.	Conductive materials only; shallow penetration; lift-off effect limitations; proximity to surface required.
PECT	Corrosion inspection under insulation/cladding without removal.	Ferromagnetic substrates only; low resolution; insensitive to micro-defects; EMI susceptibility.
UT	Wall thickness measurement, internal/external corrosion, crack detection in metallic/non-metallic pipes (chemical/power).	Requires couplant (water/gel); surface roughness compromises accuracy; unsuitable for high temperatures; limited for thin-walled pipes.
AET	Real-time monitoring of active defects (e.g., stress corrosion cracking, leaks); structural integrity assessment.	Detects dynamic defects only; low positioning accuracy; background noise interference; requires permanent sensors.
Machine Vision Inspection	Visual surface defects (cracks, deformation, deposits); weld inspection.	Lighting-dependent; lenses vulnerable to dirt/moisture; limited to line-of-sight areas; high computational load.
Laser Scanning	High-precision 3D topography (deformation, dents); internal wall corrosion imaging.	High cost; requires clean surfaces; slow scanning speed; unsuitable for high-curvature pipes.
DFOS	Long-distance leak monitoring; third-party intrusion warning; temperature/strain distribution.	Limited spatial resolution; positioning errors; complex installation/maintenance; low micro-leak sensitivity.
Wheeled/ Tracked Robots	Medium-large diameter pipes (>200 mm) with straight/gentle bends; multi-sensor payload (MFL/UT/cameras).	Poor obstacle negotiation (T-joints/diameter changes); limited traction; weak geometric adaptability.
Continuum Robots	Small-diameter (≥75 mm), multi-branch, vertical pipes; modular design navigates valves.	Extremely high cost; complex control algorithms; limited communication range; short battery endurance.
